# The Protective Effects of *Zornia diphylla* (L.) Pers. Against Acute Liver Injury Induced by Carbon Tetrachloride in Mice

**DOI:** 10.3389/fphar.2021.764282

**Published:** 2021-11-24

**Authors:** Su-Zhi Xie, Xiang-Yang Zhai, Sheng-Yan Xi, Ying-Kun Qiu, Yu-Mei Zhang, Xiang-Jun Kong, Yun-Hong Li, Lin Zhu, Zheng Wang, Shan-Gang Zhang, Shu-Qiong Huang, Da-Wei Lu, Zheng Wang

**Affiliations:** ^1^ Department of Pharmacy, Xiamen Haicang Hospital, Xiamen, China; ^2^ Department of Traditional Chinese Medicine, School of Medicine, Xiamen University, Xiamen, China; ^3^ Department of Traditional Chinese Medicine, Xiang’an Hospital of Xiamen University, Xiamen, China; ^4^ School of Pharmaceutical Sciences, Xiamen University, Xiamen, China; ^5^ Department of Pharmacy, Xiang’an Hospital of Xiamen University, Xiamen, China; ^6^ Department of Pharmacy, Zhongshan Hospital Affiliated to Xiamen University, Xiamen, China

**Keywords:** *Zornia diphyllxa* (L.) Pers., acute liver injury (ALI), traditional Chinese medicine, antioxidant enzymes, inflammatory signaling pathway

## Abstract

**Background:** *Zornia diphylla* (L.) Pers. (ZDP) is a traditional Chinese herbal medicine that has been used for several decades to treat patients with liver diseases. Whether ZDP is best administered as a single agent or adjunctive therapy has yet to be determined as does the mechanism whereby it exerts its effects on antagonizing acute liver injury (ALI).

**Aim of the study:** To investigate the protective effects of ZDP on ALI induced by carbon tetrachloride (CCl_4_) and the potential underlying mechanisms.

**Materials and Methods:** Sixty adult mice were randomized into six study groups (*n* = 10/group). Three groups were treated with different concentrations of ZDP (2.5, 1.25, 0.625 g/kg), one with bifendate (0.0075 g/kg) alone (positive control) and one with physiologic saline (normal, negative control). All groups were treated for 14 days. Two hours after the last administration, the normal group received an intraperitoneal injection of peanut oil, and the other five groups received an intraperitoneal injection of an equal dose of CCl_4_ peanut oil solution. At 24 h, the liver index, histology and serum or tissue levels and/or protein expression of aspartate aminotransferase (AST), alanine aminotransferase (ALT), total bilirubin (TBIL), alkaline phosphatase (ALP), superoxide dismutase (SOD), malondialdehyde (MDA), catalase (CAT), glutathione (GSH), Akt, phosphorylated Akt (p-Akt), nuclear factor kappa B p65 (NF-κB p65), inhibitor of NF-κB α (IκB-α), interleukin-1 β (IL-1β), interleukin-6 (IL-6), tumor necrosis factor α (TNF-α), E-cadherin and vimentin were determined.

**Results:** Compared to the model controls, the degree of inflammatory cell infiltration and hepatocyte injury of liver tissue was relieved in the bifendate and three ZDP groups; liver index in the ZDP (2.5, 1.25 g/kg) groups and serum liver function indices in the ZDP (2.5, 1.25 and 0.625 g/kg) groups were decreased; antioxidants SOD, CAT and GSH in liver tissue were increased but the lipid peroxidation index MDA was decreased; protein expression of inflammatory cytokines Akt, p-Akt, NF-κB p65, IκB-α, IL-1β, IL-6 and TNF-α in the liver was ameliorated, and E-cadherin expression was increased. The results of liver histopathology also showed that ZDP had a significant effect on ALI.

**Conclusion:** ZDP has obvious protective effects on CCl_4_-induced ALI as a single therapy and appears to act by inhibiting oxidation, reducing the release of inflammatory factors and promoting hepatocyte repair.

## Introduction

In recent years, due to the emergence of drug abuse, and the widespread use of various types of therapeutic drugs with many side effects, clinical drug-induced liver damage is more common, and to date, there is no effective prevention and treatment measures in clinical practice. Acute liver injury (ALI) refers to sudden hepatocyte damage and abnormal liver function due to various reasons within a short period of time. Drug poisoning, viral infection, immune response, and ischemia reperfusion are the most common ALI inducing factors (Hassan and Fontana, 2019; Meyerson and Naini, 2020), of which, liver injury caused by chemical poisoning is the most frequent cause of ALI, with an incidence rate of over 40% in patients over 50 years old (Lu et al., 2019). ALI can induce a series of complications and aggravate the progress of other diseases, which should be actively prevented and treated. Most patients with ALI recover following drug treatment, but some patients will progress to acute liver failure with high mortality. In the prevention and treatment of liver diseases, many types of traditional Chinese medicine (TCM) and extracts of TCM have proved to be effective in liver protection (Ye et al., 2009; Ren et al., 2019; Mo et al., 2020), and have been widely used in clinical practice. Many traditional Chinese medicines have the advantages of a good curative effect, safety and practical benefits; thus they have received considerable attention in the field of disease treatment, prevention and health care.

*Zornia diphylla* (L.) Pers. (ZDP), known as “Dingkuicao” in Chinese, is the dry whole herb of *Zornia diphylla* (L.) Pers. from the *Fabaceae* family, which grows in tropical areas, mainly in Zhejiang, Fujian, Guangxi, Yunnan and other regions in China (National Administration of Traditional Chinese Medicine of China, 1999; Wang, 2014). It is a Chinese common folk and traditional herbal medicine and is widely used. Its main effective ingredients include flavonoid glycosides (quercetin), isoflavones, phenols (kaempferol) and amino acids (Chen et al., 2012; Ren et al., 2012; Chen and Guan, 2020). In TCM theory, it is sweet in flavor and cool in nature, and acts on the spleen and liver channels, and has the effects of clearing heat and resolving toxins, removing blood stasis and relieving swelling and draining dampness of both the liver and gallbladder to relieve jaundice. It is commonly taken orally to treat fever with a wind-heat pattern, acute gastroenteritis, jaundice, acute or chronic hepatitis, dysentery, pharyngitis, conjunctivitis, mastitis, malaria, tumors and other diseases (Pharmaceutical Bureau of General Hospital of Southern Theater Command of Chinese People’s Liberation Army, 1979; National Administration of Traditional Chinese Medicine of China, 1999; Arunkumar et al., 2012; Ren et al., 2012; Govindarajan et al., 2016). Externally, it is used for the treatment of swelling and pain, sores, venomous snake bites and so on. In areas with a high incidence of hepatic diseases, ZDP is mainly used for the prevention and treatment of icteric hepatitis, with good curative effects (Wang, 2014). Therefore, we speculate that ZDP may have a therapeutic effect on inflammatory injury of the liver. Although anti-inflammatory experiments on ZDP have been carried out and clinical experience of ZDP on acute hepatitis has been confirmed (National Administration of Traditional Chinese Medicine of China, 1999; Chen et al., 2018a), there are no reports on the therapeutic effect and mechanism of ZDP in ALI.

In order to verify the hepatoprotective activity of ZDP, a carbon tetrachloride (CCl_4_)-induced ALI model was established in mice. In addition to biochemical, molecular biological protein analysis and pathomorphological observations, the hepatoprotective effect of ZDP was confirmed and its possible mechanism was discussed, which not only explains the efficacy of TCM using modern scientific theory, but also provides the necessary experimental data for further development of ZDP in the treatment of liver disease.

## Materials and Methods

### Experimental Animals

Sixty specific pathogen-free healthy ICR mice (half male and half female) aged 4–6 weeks, weighing 20 ± 2 g, were purchased from Minhou Wushi Experimental Animal Trading Co., Ltd. (Fuzhou, China), which has the Experimental Animal Production License Number: SCXK (Min) 2016-0002. All the experimental mice [Experimental Animal Usage License Number: SYXK (Min) 2018-0009] were randomly divided into six groups with ten mice in each group, and the experiment was carried out after 7 days of adaptive feeding. The room temperature was kept at 22 ± 2°C, the air humidity was controlled at 55 ± 20%, light was 12 h/day, and the mice were free to drink pure water and eat freely. The experiment was carried out in strict accordance with the standards of the Laboratory Animal Management and Ethics Committee of Xiamen University (Xiamen, China) (No. XMULAC2012-0039).

### Experimental Drugs and Medicinal Preparation

One kilogram of dried whole herb of ZDP (Voucher Number: 181012) was purchased from Guangdong Kangmei Pharmaceutical Co., Ltd., Puning, China. The voucher specimen was deposited at the Xiamen Botanical Garden (http://sweetgum.nybg.org/science/ih/herbarium-details/?irn=249232) (Herbarium Code: XMBG) for future reference. It was crushed, sieved through a 50 mesh, and weighed. ZDP powder was extracted three times with 10× distilled water, for 2 h each time, and the filtrate was concentrated and dried in a vacuum drier. The weight of the final freeze-dried powder of ZDP (50 g) was 5.3 g, and the extraction yield was 10.6%. Distilled water was used to prepare solutions containing 0.25, 0.125 and 0.0625 g of extract per ml, respectively, labeled as high, medium and low dose extracts of ZDP, and placed in a refrigerator at 4°C until used. Biphenyl diester drop pills (Lot No.: A02180903) (Specification: 15 mg × 250 pills) were purchased from Wanbang Pharmaceutical Group Co., Ltd. (Wenling, China), diluted in distilled water and an aqueous solution was prepared at the concentration of 0.75 mg/ml for the experiments.

### Main Reagents

In this study, the following reagents were used: an alanine aminotransferase (ALT) assay kit (Lot No.: C009-2-1), aspartate aminotransferase (AST) assay kit (Lot No.: C010-2-1), alkaline phosphatase (ALP) assay kit (Lot No.: A059-2-2), total bilirubin (TBIL) assay kit (Lot No.: C019-1-1), catalase (CAT) assay kit (Lot No.: A007-1), glutathione (GSH) assay kit (Lot No.: A006-2-1), malondialdehyde (MDA) assay kit (Lot No.: A003-4-1) and superoxide dismutase (SOD) assay kit (Lot No.: A001-3), which were purchased from Nanjing Jiancheng Bioengineering Research Institute Co., Ltd. (Nanjing, China). An interleukin-1 β (IL-1β) ELISA kit (Lot No.: M0037c) and tumor necrosis factor α (TNF-α) ELISA kit (Lot No.: M0049c) were purchased from Elabscience Biotechnology Co., Ltd. (Wuhan, China). Akt antibody (Lot No.: 4691), p-Akt antibody (Lot No.: 13038), nuclear factor kappa B p65 (NF-κB p65) antibody (Lot No.: 3033), inhibitor of NF-κB α (IκB-α) antibody (Lot No.: 4814), interleukin-6 (IL-6) antibody (Lot No.: 12912) and TNF-α antibody (Lot No.: 11948) were purchased from Cell Signaling Technology, Inc. (Boston, United States). IL-1β antibody (Lot No.: 16806-1-AP), E-cadherin antibody (Lot No.: 20874-1-AP) and rabbit secondary antibody (Lot No.: SA00001), were purchased from Proteintech Group, Inc. (Chicago, United States). Vimentin antibody (Lot No.: ARG65682) was purchased from Arigo Biolaboratories Corp. (Shanghai, China). A BCA kit (Lot No.: QB214754) was purchased from Thermo Fisher Scientific Technology Co., Ltd. (Shanghai, China). RIPA buffer (Lot No.: R0010) was purchased from Beijing Solarbio Science and Technology Co., Ltd. (Beijing, China). CCl_4_ solution (Lot No.: C805332) was purchased from Shanghai Macklin Biochemical Co., Ltd. (Shanghai, China).

### Main Instruments

The following instruments were employed: D3024R High-speed freezing microcentrifuge (Scilogex Corporation, Louisville, United States), GloMax GM3030 Microplate reader (Promega Corporation, Madison, United States), DXC800 Beckman Automatic Biochemistry Analyzer (Beckman Coulter, Inc., Brea, California, United States), 10RTEX-5 Oscillation apparatus and TSB-108 Shaking table (Haimen Kylin-Bell Lab Instruments Co., Ltd., Haimen, China), 041BR126545 Electrophoresis apparatus (BIO-RAD, United States), HJ-4A Magnetic constant temperature stirrer (Jintan Chengxi Zhengrong Experimental Instrument Factory, Jintan, China), HLD-10002 Electronic balance (Hangzhou Youheng Weighing Equipment Co., Ltd., Hangzhou, China), SB-1100 Water bath pot (Xiamen Jingyi Xingye Technology Co., Ltd., Xiamen, China), DK-8D Electric thermostatic bath (Shanghai Jinghong Experimental Equipment Co., Ltd., Shanghai, China), LeicaRM2016 histotome (Leica Co., Solms, Germany), Olympus CX41 Microscope (Olympus Optical Co., Ltd., Tokyo, Japan) and Thermo Q-Exactive Orbitrap Mass spectrometer equipped with a Thermo UltiMate 3000 HPLC system (Thermo Fisher Scientific, Bremen, Germany).

### High Performance Liquid Chromatography-Tandem Mass Spectrometry Analysis

The major chemical constituents of ZDP were profiled by high performance liquid chromatography (HPLC) coupled with a high resolution electrospray ionization mass detector. The freeze-dried powder of ZDP dissolved in water at a concentration of 10 mg/ml was used as the test solution. The solution was filtered with a 0.22 µm nylon filter membrane before injection into the HPLC system. The 1D-HPLC separations were performed on a Kinetex C18 column (50 × 4.6 mm i.d., 5.0 µm, Phenomenex Inc., Torrance, United States). The mobile phase was water (A) and methanol (B), gradiently eluted according to the following elution program: 0–25 min, 20% B to 100% B and 100% B was kept until 30 min. The column was maintained at 30°C and eluted at a flow rate of 0.3 ml/min. Sample loading of the test solution was 10 µl (10 mg/ml). The high resolution electrospray ionization detector was used to record the HPLC chromatograms. The MS spectra, with an *m/z* scan range from 100 to 1000, was acquired both in positive and in negative mode. The ionization process was operated with a spray voltage of 3.8 and 3.5 kV for the positive and negative mode, respectively.

### Animal Groupings, Drug Administration and Establishment of the Murine Acute Liver Injury Model

Sixty mice were divided into six treatment groups (*n* = 10/group) according to a random number table, namely, normal (negative control), model, western medicine control (biphenyl diester), and high, medium and low-dose water extracts of ZDP (ZDP-H, ZDP-M, ZDP-L, respectively). The normal and model groups were given 0.9% physiological saline by gavage, once a day. The western medicine control group was given an aqueous solution of bifendate dropping pills by gavage at 0.0075 g/kg (0.00075 g/ml), once a day. Based on clinical experience, the commonly-used daily dose of ZDP (dried whole herb) for adults with a body weight of 60 kg is 12 g (Ran, 1993). According to the study of “dose translation from animal to human studies revised” by Reagan-Shaw et al., the human equivalent dose (HED) (mg/kg) = animal dose (mg/kg) × Animal *Km*/Human *Km* (Reagan-Shaw et al., 2007). The mouse dose of ZDP (g/kg) = 12 g/60 kg × 37 (Human *Km*)/3 (Mouse *Km*) = 2.5 g/kg, which was used in the ZDP high-dose group in this study. The three ZDP groups were given the aqueous extract solution of ZDP by gavage at 2.5 g/kg (0.25 g/ml), 1.25 g/kg (0.125 g/ml) and 0.625 g/kg (0.0625 g/ml), respectively, once a day. Each group was treated for 14 consecutive days. CCl_4_ solution was prepared into a 0.1% peanut oil diluent. Two hours after the last administration, mice in the normal control group were intraperitoneally injected with an equal dose of peanut oil (10 ml/kg), whereas mice in the other five groups were intraperitoneally injected with CCl_4_ peanut oil solution (concentration of 0.5%, 10 ml/kg) to induce the murine model of ALI (Wang et al., 2014; Cui et al., 2019). The changes in serum ALT and AST were determined, and a pathomorphological examination of the liver was carried out. When the levels of ALT and AST in the model group were more than five times higher than those in the normal group, and the pathological features of the liver included necrosis and fatty degeneration in the central region of the lobules, successful establishment of the ALI model was confirmed.

Sixteen hours after modeling, food and water consumption was prevented in all mice. At 24 h, the mice in each group were weighed, the eyeballs were removed, blood was collected to prepare serum and the liver tissue was then removed after cervical dislocation. The liver tissue was washed twice with phosphate-buffered saline (PBS). After weighing, half of the liver tissue was routinely fixed and embedded in wax blocks, and the other half was stored at −80°C.

### Determination of Serum AST, ALT, TBIL and ALP

Using a sterile coagulation promoting tube, peripheral blood (0.8 ml) was collected from the orbital sinus of each mouse prior to sacrifice. After standing for 2 h at room temperature, the blood samples were centrifuged at 3,000 g for 10 min. The supernatant was collected and stored in a refrigerator at −80°C. Serum ALT, AST, TBIL and ALP levels were determined using their respective assay kits and the automatic biochemistry analyzer according to the manufacturer’s instructions.

### Determination of SOD, MDA, CAT, GSH, IL-1β and TNF-α Expression in Hepatic Tissue

At the time of sacrifice, some of the hepatic tissue not required for histology was washed with PBS, cut into small pieces using ophthalmic scissors, transferred to a homogenizer, exposed to protein extraction solution, incubated in an ice bath and then centrifuged at 15,000 g. The supernatants were collected and stored at −20°C for protein quantification. SOD, MDA, CAT, GSH, IL-1β and TNF-α ELISA kits were used to determine the concentrations of these parameters according to the manufacturer’s instructions (Chen et al., 2007). The detection steps were as follows: 300 μl washing solution was added to soak the enzyme plate for 30 s, the washing solution was then discarded, the micropores were patted dry on absorbent paper, 50 μl of detection buffer was added and 50 μl of 2× diluted standard solution was added to the standard well. Test buffer (50 μl) and 50 μl of standard diluent were added to the blank well, 80 μl of test buffer and 20 μl of standard diluent were added to the sample wells containing the blood samples: 50 μl of diluted detection antibody was added to each well and incubated at room temperature for 1.5 h, the washing solution was discarded, 300 μl of washing solution was added to each well and the plate was washed six times and patted dry; 100 μl of diluted horseradish peroxidase labeled streptavidin was added to each well and incubated at room temperature for 30 min, step 5 was repeated, 100 μl of chromogenic substrate was added to each well and incubated in the dark for 5–30 min, and then 100 μl was added to each well within 30 min. The OD value at 450 nm was detected by the enzyme reader.

### Analysis of the Liver Index

Following blood collection, the mice were immediately sacrificed by cervical dislocation. The whole liver was completely removed and placed on sterile ice. After washing with normal saline, filter paper was used to remove excess saline. The liver was weighed and the value recorded. The liver index (liver weight/body weight × 100%) was calculated (Liu et al., 2020).

### Pathological Observations of Liver Tissue

Liver tissue 1.0 cm × 1.0 cm × 0.3 cm in size was quickly cut and fixed in 4% paraformaldehyde for 24 h. A tissue block 0.4 cm × 0.4 cm × 0.3 cm in size was cut with a blade and placed into a disposable plastic embedding frame, and then dehydrated in low to high concentration ethanol. After dehydration, the tissue was made transparent with xylene. The embedded wax blocks were sliced into 5 μm wax strips, spread on the water surface at 40–45°C and baked in an incubator for 2 h at 60°C. The paraffin strips were dewaxed with xylene and washed with ethanol and distilled water. Following hematoxylin and eosin (HE) staining, the stained sections were dehydrated using a low to high concentration ethanol gradient, and xylene added; the sections were removed, excess xylene was wiped off and a drop of 50% neutral gum was added. The slides were covered and sealed using neutral balsam. The pathological changes in the tissues were observed under a light microscope and recorded.

### Measurement of AKT, p-AKT, NF-κB p65, IκB-α, IL-1β, TNF-α, IL-6, E-Cadherin and Vimentin Protein Expression in Hepatic Tissues by Western Blot

Liver tissue (30 μg) from each sample was used for protein analysis. The detection steps were as follows: 4 × loading buffer was added, the samples were placed in a boiling water bath for 10 min, centrifuged at 10,000 rpm for 10 min; 10% or 12% SDS-polyacrylamide gel electrophoresis (SDS-PAGE) was prepared according to the molecular weight of protein for electrophoretic separation of protein samples; the proteins on SDS-PAGE were transferred to a polyvinylidene fluoride (PVDF) membrane by electric transfer; after electric transfer, the PVDF was immersed in sealing solution containing 5% nonfat dry milk and incubated for 1 h at room temperature. Phosphate buffer solution-tween-20 (PBST) was used to wash the membrane three times for 5 min each time; the primary antibody of Akt (1: 1000), p-Akt (1: 4000), NF-κB p65 (1: 1000), IκB-α (1: 1000), IL-1β (1: 1000), IL-6 (1: 1000), TNF-α (1: 1000), E-cadherin (1: 1000) or vimentin (1: 1000) was added, respectively, then incubated overnight at 4°C. After washing 3 times with PBST for 20 min each time, horseradish peroxidase labeled secondary antibody solution (1: 10,000) was added, and incubated at room temperature for 1 h. After washing three times with PBST for 10 min each time, the PVDF membrane was reacted with fresh enhanced chemiluminescence solution for 2 min, and quickly exposed in a dark room (Zhang et al., 2016). The optical density values of the developing target strip and GAPDH reference strip were analyzed by ImageJ software.

### Statistical Analysis

Data are expressed as mean ± standard deviation (SD) ( $\bar{x}$ ± s). Statistical analyses were performed using GraphPad Prism 7.0 (GraphPad Software Inc., La Jolla, United States). One-way analyses of variance (One-Way ANOVA) followed by Tukey’s post-test was used to compare the means of multiple groups. The LSD (least significant difference) method was selected for post-hoc analysis, and the Kruskal-Wallis non-parametric H test was applied when there was one inconsistency. *p* < 0.05 was considered statistically significant.

## Results

### Analysis of ZDP

**FIGURE 1:**
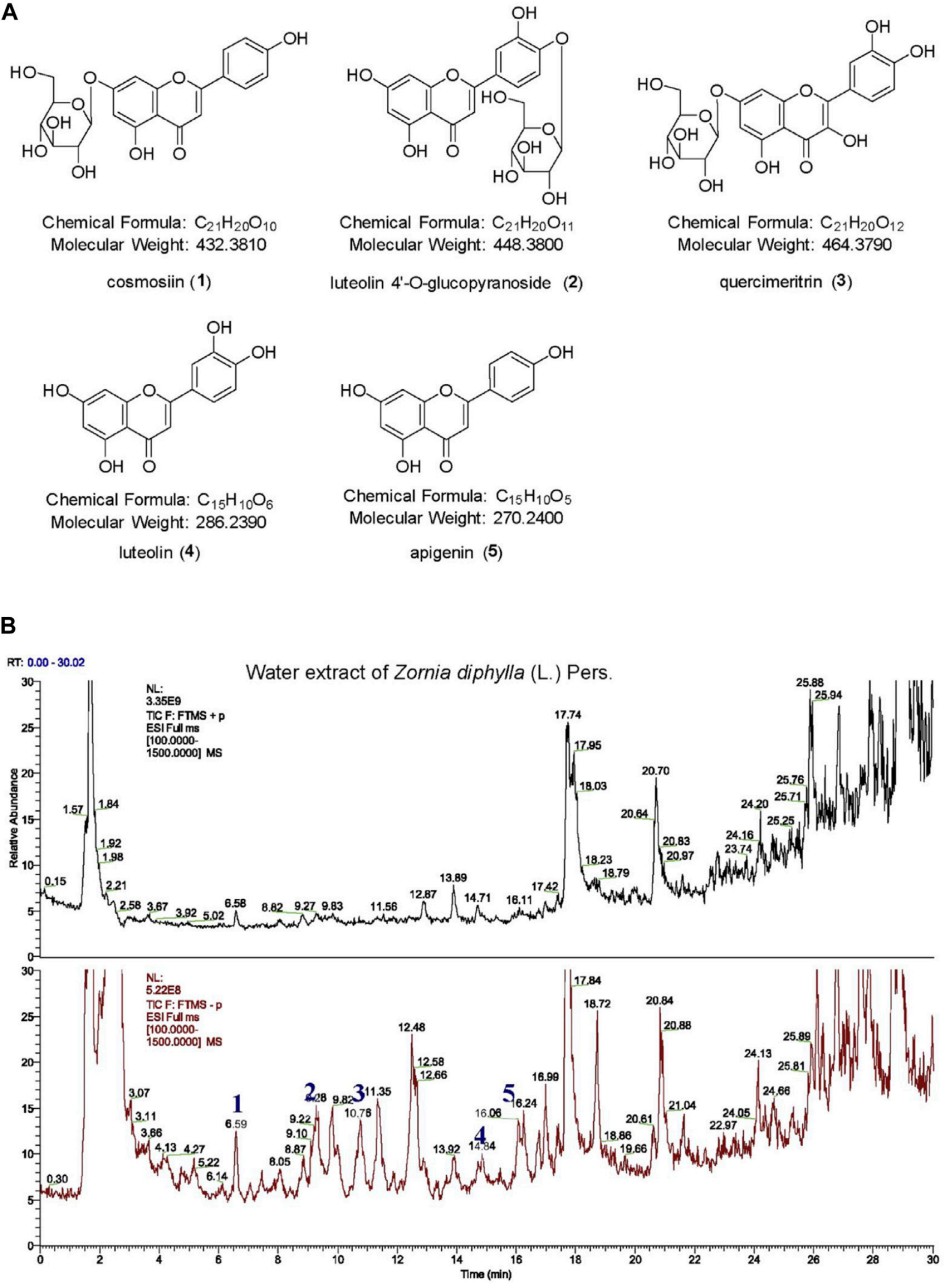
HPLC-HR ESI MS analysis of a water extract from the herb of *Zornia diphylla* (L.) Pers. **(A)** Chemical structures of the major identified components of ZDP: cosmosiin (1), luteolin 4′-O-glucopyranoside (2), quercimeritrin (3), luteolin (4) and apigenin (5). **(B)** HPLC-MS spectra fingerprint of ZDP. Upper: positive mode (+); lower: negative mode (−).

The water extract of ZDP was separated using the HPLC-MS system and its chromatographic fingerprinting was established. Comparing the retention time (Rt), ultraviolet and mass spectra with reference samples, the following five major components were identified: cosmosiin (peak 1, Rt = 6.59 min), luteolin 4′-O-glucopyranoside (peak 2, Rt = 9.28 min), quercimeritrin (peak 3, Rt = 10.76 min), luteolin (peak 4, Rt = 14.84 min) and apigenin (peak 5, Rt = 16.06 min) as shown in Figure 1. The detailed results of the components of ZDP identified by HPLC are shown in Table 1.

**TABLE 1:** Components of the water extract of ZDP from the HPLC-HR ESI MS spectra.

No	Name	FW	MW	tR (min)	[M-H]^-^	[M+H]^+^
1	cosmosiin	C_21_H_20_O_10_	432	6.59	431.1199	433.1336
2	luteolin 4′-O-glucopyranoside	C_21_H_20_O_11_	448	9.28	447.0937	449.1080
3	quercimeritrin	C_21_H_20_O_12_	464	10.76	463.0892	465.1022
4	luteolin	C_15_H_10_O_6_	286	14.84	285.0404	287.0550
5	apigenin	C_15_H_10_O_5_	270	16.06	269.0452	271.0596

### Effects of ZDP on the Pathology of Liver Tissues

**FIGURE 2:**
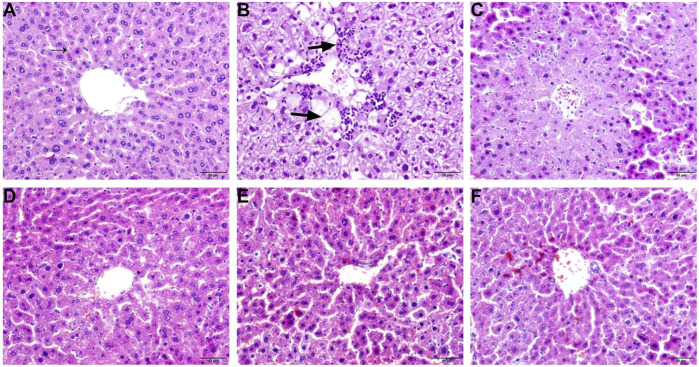
ZDP ameliorates pathological changes of liver tissues in the CCl_4_-induced murine model of ALI. Sections were stained with HE and viewed at a magnification of ×400. Scale bar = 50 μm. **(A)** Normal control; **(B)** Model control; **(C)** Bifendate control (0.0075 g/kg) (positive control); **(D)** ZDP-L (0.625 g/kg); **(E)** ZDP-M (1.25 g/kg); **(F)** ZDP-H (2.5 g/kg).

To investigate the therapeutic effects of ZDP on the pathological changes due to ALI, HE staining was performed. As shown in Figure 2, the HE-staining results showed that the liver cells in the normal (negative control) group were arranged normally, the cytoplasm was even, the nucleus and nucleolus were obvious and the central vein was clearly visible (Figure 2A). The structure of the liver cells in the model group was seriously damaged, the central zone of the lobule showed coagulated necrosis, the surrounding liver tissue showed vacuolar degeneration, some cell necrosis, nuclear shrinkage, granular degeneration and a large number of inflammatory cells had infiltrated around the central vein (as shown by the arrow in Figure 2B). The liver cells in the bifendate treated control group and the three ZDP-treated groups were relatively intact, with a small number of inflammatory cells (Figures 2C–F), and pathological damage was significantly improved compared with that in the model group. Compared with the model group, in the three ZDP-treated groups the infiltration of inflammatory cells was reduced, the degree of liver cell injury was reduced to a large extent and the effect was more obvious than that in the bifendate treated control group.

### Effects of ZDP on Liver Index, Wet Liver Weight and Body Mass

**FIGURE 3:**
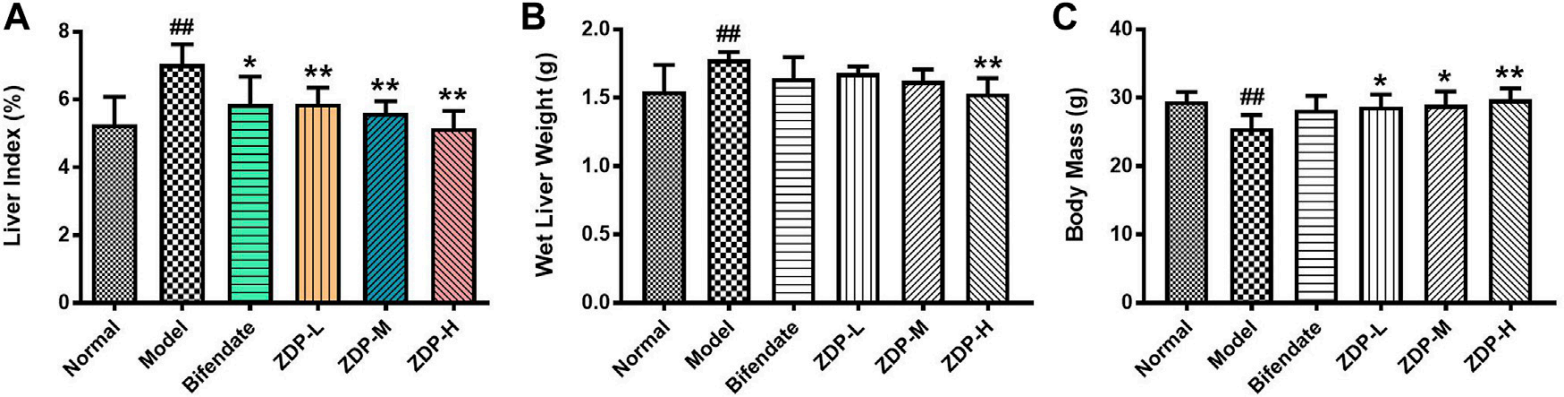
ZDP decreases the liver index, wet liver weight and body mass in the murine model of ALI induced by CCl_4_. Liver index **(A)**, wet liver weight **(B)** and body mass **(C)** were measured in mice (Normal control, Model control, Bifendate-treated control, ZDP-L, ZDP-M and ZDP-H). Data from six to eight mice per group are expressed as the mean ± SD. Statistical analysis: ^##^ *p* < 0.01 compared with the normal group; *p < 0.05, **p < 0.01 compared with the model group, One-Way ANOVA.

Changes in body mass and liver index can reflect the degree of liver injury (Li et al., 2019). As shown in Figure 3, CCl_4_ significantly reduced the body mass of mice (*p* < 0.01). Compared with the model group, the intake of ZDP significantly slowed the decrease in mouse body mass (*p* < 0.01 or *p* < 0.05), indicating that ZDP could inhibit the decrease in body mass caused by CCl_4_. Compared with the normal group, the liver index of mice in the model group was significantly increased (*p* < 0.01). Compared with the model group, the liver index of mice in the bifendate-treated positive control group and three ZDP groups was significantly decreased (*p* < 0.01 or *p* < 0.05), but there was no statistically significant difference in the liver index among the ZDP dose groups (*p* > 0.05).

### Effects of ZDP on Liver Function Indices (Serum AST, ALT, TBIL and ALP)

**FIGURE 4:**
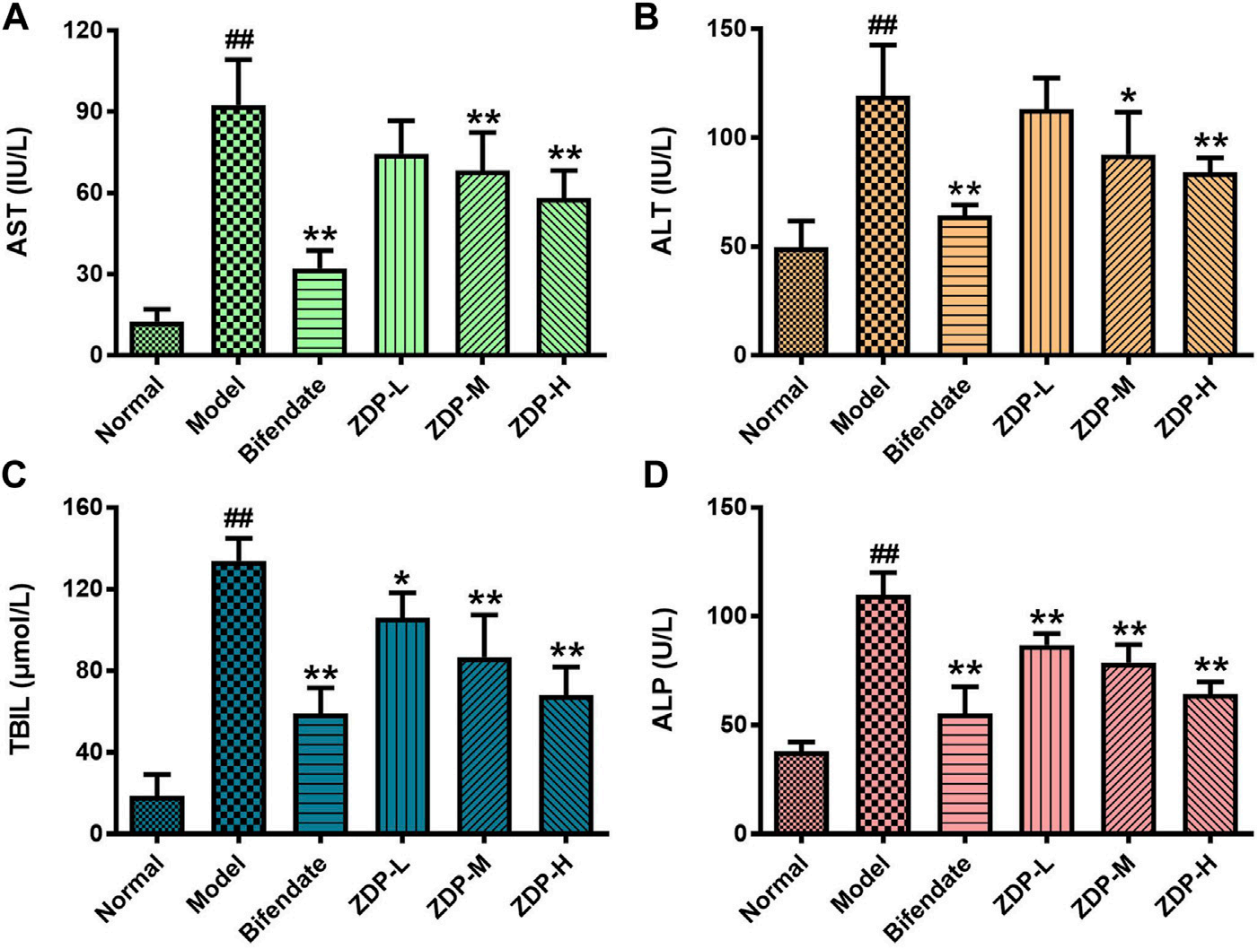
ZDP decreases the activities of serum AST, ALT, TBIL and ALP in the murine model of ALI induced by CCl_4_. AST **(A)**, ALT **(B)**, TBIL **(C)** and ALP **(D)** activities were measured in mouse serum samples (Normal control, Model control, Bifendate-treated control, ZDP-L, ZDP-M and ZDP-H) by ELISA. Data from six to eight mice per group are expressed as the mean ± SD. Statistical analysis: ^##^ *p* < 0.01 compared with the normal group; *p < 0.05, **p < 0.01 compared with the model group, One-Way ANOVA.

The increase in ALT reflected the damage to hepatocyte membranes. The increase in AST indicated hepatocyte organelle damage. AST, ALT, TBIL and ALP are the most direct and important indicators of liver damage and show whether therapeutic agents have a protective effect against liver injury (Crismale and Friedman, 2020; Owojuyigbe et al., 2020). As shown in Figure 4, compared with the normal group, the serum AST, ALT, TBIL and ALP activities in the model group were significantly increased (*p* < 0.01). Compared with the model group, the serum AST and ALT activities in mice in the ZDP group treated with 2.5 or 1.25 g/kg and in the positive control (Bifendate-treated) group were significantly decreased (*p* < 0.01 or *p* < 0.05), and the activities of TBIL and ALP in the bifendate-treated group and all ZDP groups were also obviously decreased (*p* < 0.01). However, there was no significant difference in the above indices between the three ZDP groups (*p* > 0.05). These results show that ZDP repaired the damage to the liver cell membrane and organelles induced by CCl_4_, and liver function was significantly improved.

### Effects of ZDP on Hepatic Antioxidant Activity (SOD, MDA, CAT and GSH Content)

**FIGURE 5:**
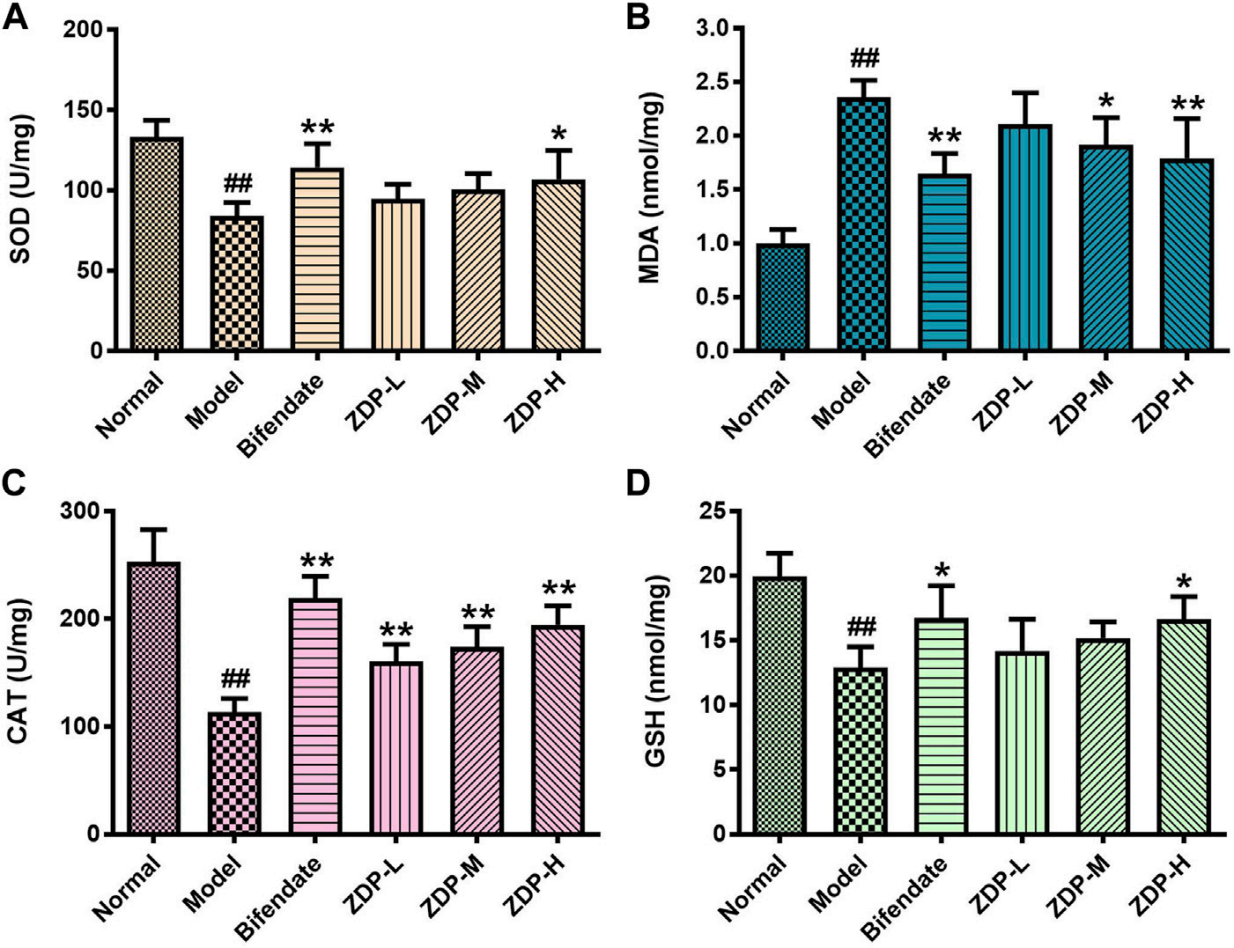
Effects of ZDP on the activity of liver SOD, MDA, CAT and GSH in the murine model of ALI induced by CCl_4_. SOD **(A)**, MDA **(B)**, CAT **(C)** and GSH **(D)** activities were measured in hepatic tissue samples (Normal control, Model control, Bifendate-treated control, ZDP-L, ZDP-M and ZDP-H) by ELISA. Data from six to eight mice per group are expressed as the mean ± SD. Statistical analysis: ^##^ *p* < 0.01 compared with the normal group; *p < 0.05, **p < 0.01 compared with the model group, One-Way ANOVA.

Oxidative stress induced by reactive oxygen species is the common pathophysiological basis in a variety of liver injuries. MDA, SOD, CAT and GSH can reflect the degree of lipid peroxidation and oxidative stress (Albrahim and Alonazi, 2021). As shown in Figure 5, compared with the normal group, the content of MDA in liver tissue of the murine model with acute liver injury induced by CCl_4_ increased, and the contents of SOD, CAT and GSH decreased (*p* < 0.01 or *p* < 0.05). Compared with the model group, the activity of SOD, the contents of CAT and GSH in the ZDP high dose group and bifendate-treated group were increased and the content of MDA was decreased (*p* < 0.01 or *p* < 0.05). The content of CAT in the ZDP low and medium dose groups was also increased significantly compared with the model group (*p* < 0.01). CCl_4_ increased the level of oxidative stress and reduced the antioxidant capacity of the liver (*p* < 0.01). These results show that ZDP can inhibit the degree of lipid peroxidation injury induced by CCl_4_ and improve the antioxidant capacity of mice.

### Effects of ZDP on the Content of Inflammatory Cytokines IL-1β and TNF-α in Hepatic Tissues

**FIGURE 6:**
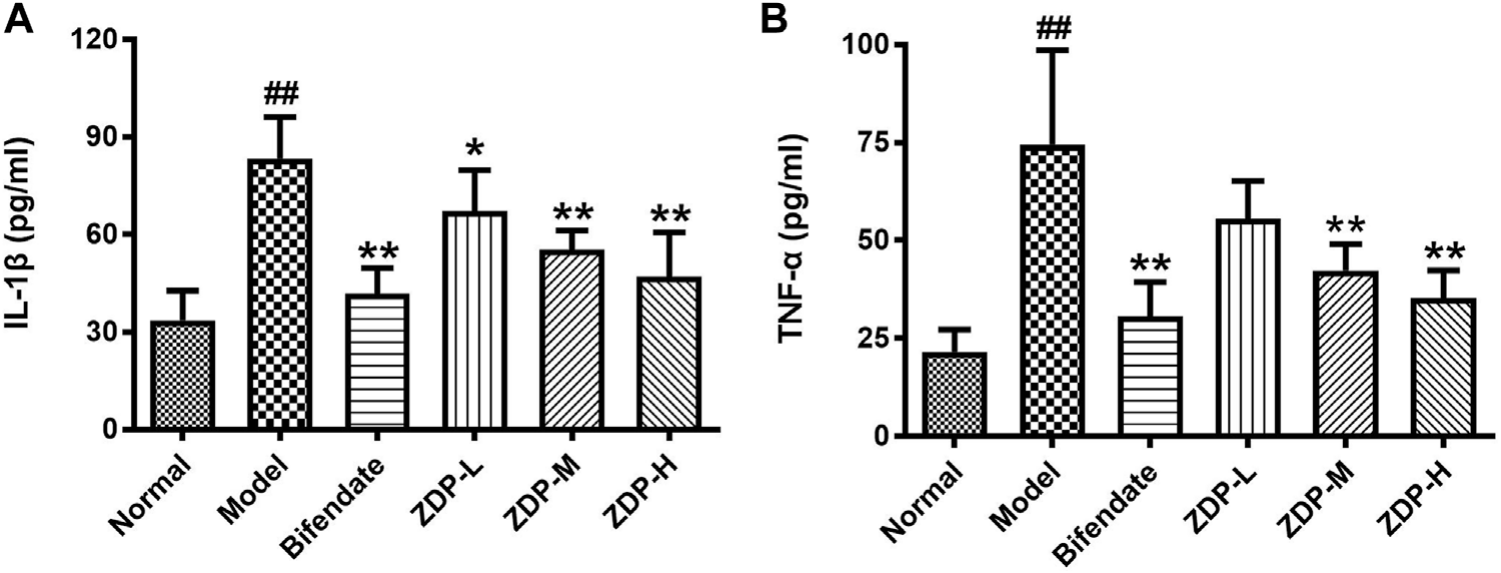
ZDP decreases the level of liver IL-1β and TNF-α in the murine model of ALI induced by CCl_4_. IL-1β **(A)** and TNF-α **(B)** levels were measured in mouse hepatic tissue samples (Normal control, Model control, Bifendate-treated control, ZDP-L, ZDP-M and ZDP-H) by ELISA. Data from six to eight mice per group are expressed as the mean ± SD. Statistical analysis: ^##^ *p* < 0.01 compared with the normal group; *p < 0.05, **p < 0.01 compared with the model group, One-Way ANOVA.

IL-1β and TNF-α are common indices used to detect the inflammatory response (Fu et al., 2020). As shown in Figure 6, compared with the normal control group, the levels of inflammatory cytokines IL-1β and TNF-α in liver tissue of the murine model of ALI induced by CCl_4_ were significantly elevated (*p* < 0.01). Compared with the model group, the level of TNF-α and IL-1β in liver tissue of mice in the medium and high dose ZDP groups and the bifendate-treated group decreased (*p* < 0.01).

### Effects of ZDP on the Expression of Inflammatory Related Proteins AKT, p-AKT, NF-κB p65, IκB-α, IL-1β, TNF-α and IL-6 in Hepatic Tissues

**FIGURE 7:**
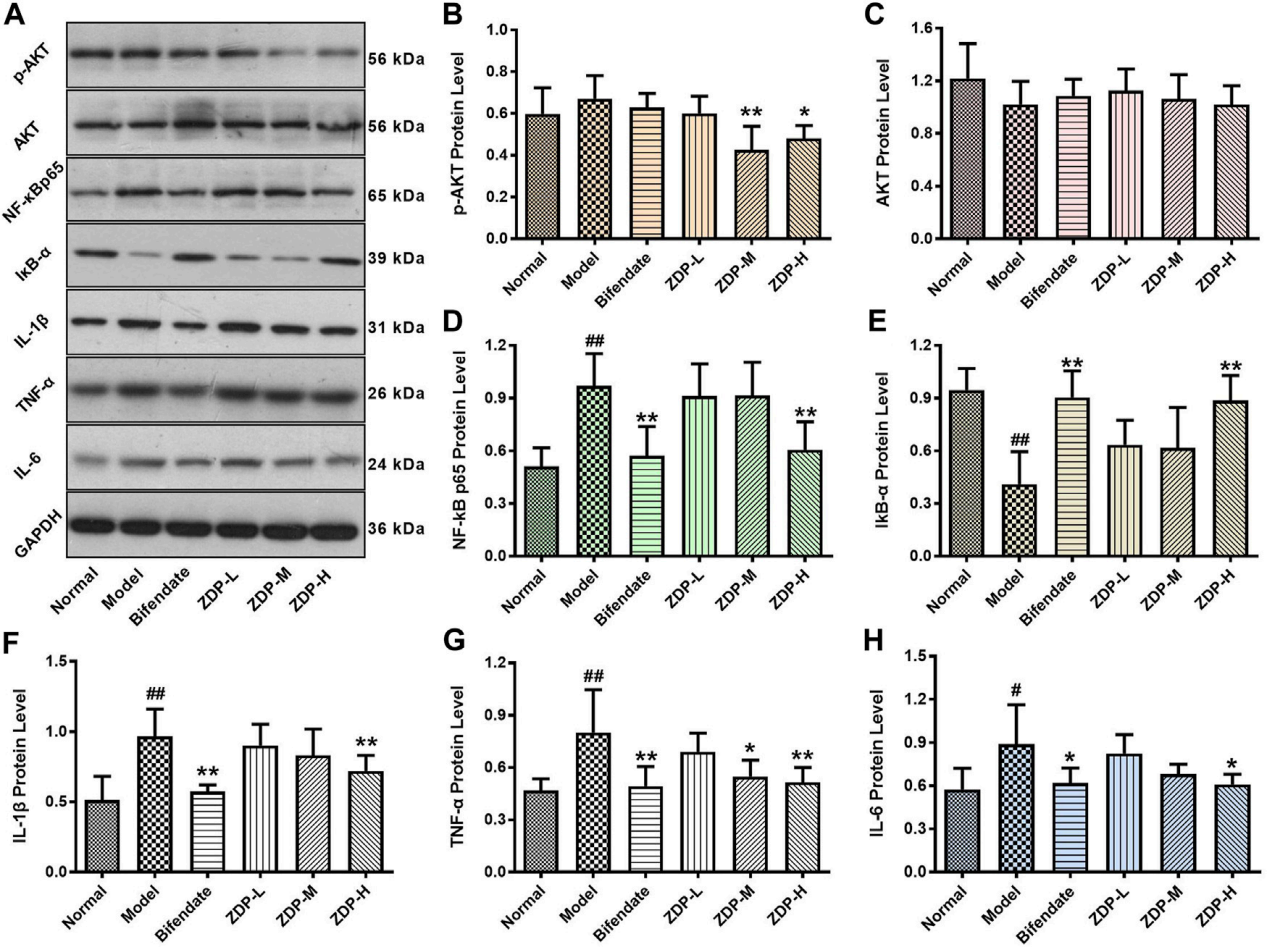
Effects of ZDP on the protein expression of Akt, p-Akt, NF-κB p65, IκB-α, IL-1β, TNF-α and IL-6 in hepatic tissues of the murine model of ALI induced by CCl_4_. **(A)** Equal protein quantities in lysates from the liver tissues of mice (Normal control, Model control, Bifendate-treated control, ZDP-L, ZDP-M and ZDP-H) were subjected to western blot with antibodies against Akt, p-Akt, NF-κB p65, IκB-α, IL-1β, TNF-α, IL-6 and GAPDH. p-Akt **(B)**, Akt **(C)**, NF-κB p65 **(D)**, IκB-α **(E)**, IL-1β **(F)**, TNF-α **(G)** and IL-6 **(H)** protein intensities were quantified by densitometry, and normalized to respective GAPDH levels. Data from six mice per group are expressed as the mean ± SD. Statistical analysis: ^#^ *p* < 0.05, ^##^ *p* < 0.01 compared with the normal group; *p < 0.05, **p < 0.01 compared with the model group, One-Way ANOVA.

The pathogenesis of ALI is closely related to oxidative stress and inflammation (Al-Dossari et al., 2020). During the process of ALI, there are three main pathways (NF-E2 related factor 2/Kelch-like ECH-associated protein 1, Toll-like receptor-4 and NOD-like receptor protein 3) that play an important role, but these pathways all involve the signals of NF-κB (Yue and Chen, 2019). The inflammatory response-related Akt-NF-κB signaling pathway plays an important role in the development of liver injury (Zhang et al., 2016; Lin et al., 2018). As shown in Figure 7, compared with the normal control group, the protein expression level of IκB (inhibitor of NF-κB) was significantly decreased (*p* < 0.01) and the levels of NF-κB, IL-1β, TNF-α and IL-6 were elevated (*p* < 0.01 or *p* < 0.05) after CCl_4_ treatment. Compared with the model group, IκB protein expression level in both the ZDP high dose group and bifendate-treated group was increased (*p* < 0.01). However, there was no significant difference in the indices of Akt and IκB between the bifendate-treated group and the three ZDP groups (*p* > 0.05). Compared with the model group, protein expression levels of NF-κB, IL-1β, TNF-α and IL-6 in the ZDP high dose group and bifendate control group were obviously decreased (*p* < 0.01 or *p* < 0.05) as well as phosphorylated Akt (p-Akt) protein expression levels in both the ZDP high and medium dose groups. These results show that ZDP can inhibit the Akt-NF-κB pathway and its downstream inflammatory factors to relieve hepatic inflammatory activity in mice.

### Effects of ZDP on the Expression of Tissue Repair Related Proteins E-Cadherin and Vimentin in Hepatic Tissues

**FIGURE 8:**
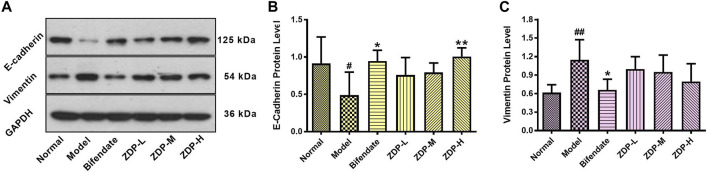
Effects of ZDP on protein expression of E-cadherin and Vimentin in hepatic tissues of the murine model of ALI induced by CCl_4_. **(A)** Equal protein quantities of lysates from mouse liver tissues (Normal control, Model control, Bifendate-treated control, ZDP-L, ZDP-M and ZDP-H) were subjected to Western blot with antibodies against E-cadherin, Vimentin and GAPDH. E-cadherin **(B)** and Vimentin **(C)** protein intensities were quantified by densitometry, and normalized to respective GAPDH levels. Data from six mice per group are expressed as the mean ± SD. Statistical analysis: ^#^ *p* < 0.05, ^##^ *p* < 0.01 compared with the normal group; *p < 0.05, **p < 0.01 compared with the model group, One-Way ANOVA.

Acute liver disease is associated with ample re-modeling of liver parenchyma leading to functional impairment (Hempel et al., 2015). Hepatocyte E-cadherin and vimentin play an important role in epithelial mesenchymal transition (EMT) and hepatic structural change to affect the repair of liver tissue injury (Zou, 2008; Zhou, 2016). As shown in Figure 8, following CCl_4_ administration, the E-cadherin protein expression levels were decreased (*p* < 0.05), but expression levels of vimentin were significantly increased (*p* < 0.01). Compared with the model group, E-cadherin protein expression in the ZDP high dose group and bifendate control group was higher (*p* < 0.01 or *p* < 0.05), and vimentin protein expression in the three ZDP groups also showed a downward trend. However, there was no significant difference in these three indices between the three ZDP groups and bifendate control group (*p* > 0.05). These results showed that ZDP had an inhibitory effect on EMT and promoted the repair of damaged hepatocytes.

## Discussion

**FIGURE 9:**
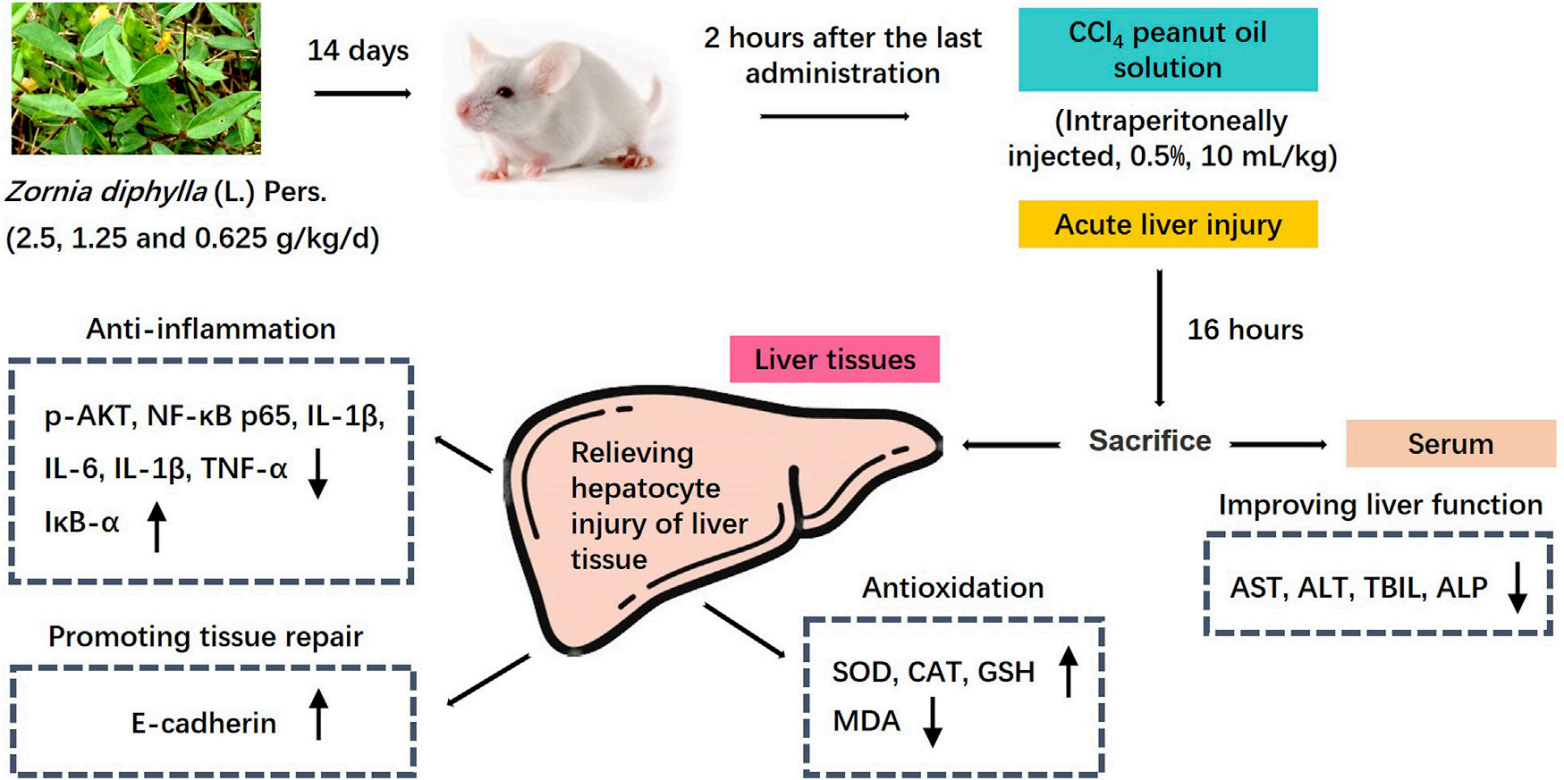
Mechanism diagram of the protective effects of ZDP on the murine model of ALI induced by CCl_4_.

The liver is the largest parenchymal organ in the human body, and its functions include catabolism, toxin removal, oxidation resistance and so on. However, the liver is easily damaged by various internal and external pathogenic factors. Liver disease is common and has been the focus of global medical attention and research, and ALI is the starting point of almost all liver diseases (Yue and Chen, 2019). Animal models of liver injury can be replicated by chemical, alcohol, immune, and biological methods. The establishment of an ALI mouse model using CCl_4_ in this study is a classic modeling method, as the symptoms, liver function indices and pathological changes are similar to human liver injury (Lin et al., 2014). CCl_4_ is administered to animals by intraperitoneal injection, and can directly enter hepatocytes. After biotransformation by liver cytochrome P450 (CYP450), CCl_3_-and CCl_3_O_2_- free radicals are generated *in vivo*. CCl_3_-binds freely with intracellular macromolecules, resulting in the destruction of hepatocyte membrane structure and function, and finally hepatocyte degeneration and necrosis (Gao et al., 2017; Chen et al., 2018b). In this study, we confirmed that the main hepatoprotective effects of the water extract of *Zornia diphylla* (L.) Pers. in CCl_4_-induced ALI in mice, and revealed that the main mechanisms underlying its efficacy were antioxidation, downregulation of the expression of pro-inflammatory signal factors and the promotion of liver tissue repair (Figure 9). These findings indicate that ZDP may be a promising therapeutic agent in the treatment of liver injury.

Following hepatocyte injury, the soluble enzymes AST and ALT present in the cytoplasm enter the blood, resulting in a significant increase in the levels of these enzymes in serum (Lian et al., 2020). This is also accompanied by an increase in TBIL, ALP, bilirubin and so on. Therefore, serum biochemical indices ALT, AST, TBIL and ALP are important in determining liver function impairment (Nwidu et al., 2018). In the present study, we found that the levels of serum ALT and AST in the model group were significantly higher than those in normal mice. Combined with the pathological results, these findings suggested that the ALI model was successfully established. Intragastric administration of different doses of ZDP reduced these indices, reduced the liver index and significantly ameliorated liver lesions, which indicated that ZDP has a significant protective effect on ALI induced by CCl_4_.

The pathogenesis of ALI is closely related to oxidative stress and the inflammatory response. Lipid peroxidation is an important mechanism of liver injury. SOD is the main antioxidant enzyme that scavenges free radicals and inhibits free radical reactions *in vivo*. The higher the activity of SOD *in vivo*, the faster the free radical scavenging rate. When hepatocytes are attacked by free radicals, SOD can be reduced due to its excessive consumption. The reduction in its activity can lead to the accumulation of oxygen free radicals in the body, resulting in peroxidation of plasma membrane, damage to the membrane structure and function, and finally to the death of hepatocytes (Sun et al., 2010; Saeedi Saravi et al., 2016; Paulino et al., 2020). MDA is a special marker of oxidative damage. Its level can directly reflect the degree of lipid peroxidation and indirectly reflect the degree of cell damage caused by free radicals (Chen et al., 2007; Abdel-Kawy 2021). SOD can remove high concentrations of superoxide anion free radicals in cells. GSH and CAT are important antioxidants and free radical scavengers *in vivo*, which can convert harmful substances in cells into harmless substances and eliminate them (Li et al., 2015; Zhao et al., 2017a). In this study, following CCl_4_ liver injury in mice, the content of MDA increased significantly and the activities of SOD, GSH and CAT decreased significantly, indicating that the ability of hepatocytes to scavenge free radicals decreased. However, the content of MDA decreased significantly and the activities of SOD, GSH and CAT increased significantly in the liver tissue of mice in the ZDP groups, suggesting that ZDP has a certain antioxidant effect and can effectively improve the ability of cells to scavenge free radicals and reduce the damage to hepatocytes under free radical attack, which may be related to the antioxidant effect. These results were consistent with those of previous research (Li et al., 2019; Ren et al., 2019).

IL-1β, IL-6, TNF-α and other inflammatory factors play an important role in the inflammatory process of acute tissue injury (Chalasani et al., 2008). In ALI, the liver cannot effectively remove endotoxin, and will produce a series of immune responses. With the largest number of macrophages in the body, Kupffer cells secrete a large number of inflammatory cytokines such as TNF-α, IL-1β and IL-6, which are involved in the occurrence of liver injury. TNF-α is the earliest inflammatory cytokine released when hepatic macrophages are damaged. IL-1β, as the strongest inflammatory factor in the body, is the product of the TNF-α cascade reaction, and is also an important mediator of the immune and inflammatory response, which promotes the expression of inflammatory cytokines and induces the cellular immune response. IL-6 is an important lymphokine, which participates in the immune response by activating and regulating immune cells. When overexpressed, IL-6 mediates the chemotaxis of inflammatory factors, exacerbates the waterfall-like inflammatory response, and finally leads to tissue damage (Triantafyllou et al., 2018). Akt (protein kinase B), a serine/threonine protein kinase, is involved in important inflammatory responses, and the activation of Akt can activate IκB kinases (IκKs), lead to the phosphorylation and degradation of IκBα, and then activate the NF-κB signaling pathway, further enhancing NF-κB expression and leading to inflammatory responses and cytokine production (Tang et al., 2016; Rahmani et al., 2020). NF-κB is a classical inflammatory factor in the immune inflammatory response. Resting NF-κB and its inhibitor IκB combine to form a complex, which allows NF-κB to remain in the cytoplasm in an inactive state with a low expression level. When hepatocytes are injured by CCl_4_, interleukin, as an important inflammatory mediator and immunomodulatory factor, activates inhibitory kappa B kinase, degrades IκB protein, dissociates NF-κB from IκB, and NF-κB then translocates from the cytoplasm to the nucleus and binds to the specific promoter, mediates the mutual expression of inflammatory cytokines within and between cells, multidirectionally regulates the inflammatory response and causes the cascade amplification effect of the inflammatory response (Dong and Xue, 2010; Luedde and Schwabe, 2011; Zhao et al., 2017b). In this study, the levels of p-Akt, NF-κB, TNF-α, IL-1β and IL-6 in liver tissue of model group mice were significantly increased, while ZDP reduced the protein expression of p-Akt, NF-κB, TNF-α, IL-1β and IL-6, which suggests that ZDP may inhibit the phosphorylation of AKT, and decrease the release of NF-κB, TNF-α, IL-1β and IL-6 to protect the liver.

Vimentin, as a type III intermediate silk protein, anchors and supports organelles in the cytoplasm of mesenchymal cells. The expression of vimentin is up-regulated during EMT (Serrano-Gomez et al., 2016). CCl_4_ can activate hepatic stellate cells, which can express vimentin in large quantities, and then start the process of liver fibrosis (Herbst et al., 1997). Our results showed that the expression of vimentin protein increased after CCl_4_ administration, and decreased in the ZDP groups, but no statistically significant difference was observed. The reason for this may be that the time of ALI was relatively short and fibrosis had not yet occurred. Cell adhesion plays an important role in cell function. It is closely related to cell growth, differentiation, tissue repair, cell damage and stability of the internal environment. E-cadherin is the most common type of cadherin, which is expressed between epithelial cells (Biswas, 2020). Although it plays an important role in tumor metastasis, there are still few reports on its relationship with tissue damage repair. Our study found that after CCl_4_ administration, the expression of E-cadherin in mouse liver decreased significantly, which was similar to that reported in other studies (Zou, 2008). In the ZDP high dose group, the expression of E-cadherin protein was significantly increased, indicating that there was a certain correlation between liver injury caused by CCl_4_ and the expression of E-cadherin. It is speculated that ZDP may improve the adhesion between cells and promote the repair of injured hepatocytes by promoting the expression of E-cadherin protein.

Due to many common challenges existing in phytopharmacological research (Heinrich et al., 2020), and the complexity of the ingredients in ZDP, there are some limitations in this study. First, as indicated above, it remains to be determined which ingredient(s) in ZDP was responsible for relieving ALI. Second, whether ZDP is effective in other ALI models remains to be determined. Finally, although encouraging, the beneficial effects of ZDP in this animal model of ALI may not be consistent with that in humans.

## Conclusion

ZDP can effectively relieve the degree of ALI induced by CCl_4_ in mice, improve the antioxidant capacity of liver cells, reduce the expression of inflammatory factors, promote liver tissue repair, and has a protective effect on CCl_4_-induced ALI in mice. Its protective effect may be related to inhibition of the activation of the inflammatory response-related Akt-NF-κB pathway.

## Data Availability

The original contributions presented in the study are included in the article, further inquiries can be directed to the corresponding author.

## Abbreviations

ALI, acute liver injury; ALP, alkaline phosphatase; AST, aspartate aminotransferase; ALT, alanine aminotransferase; CAT, catalase; EMT, epithelial mesenchymal transition; GSH, glutathione; HE, hematoxylin and eosin; HPLC, high performance liquid chromatography; IL-6, interleukin-6; IL-1β, interleukin-1 β; IκB-α, inhibitor of NF-κB α; MDA, malondialdehyde; NF-κB, nuclear factor kappa B; One-Way ANOVA, one-way analyses of variance; SOD, superoxide dismutase; TBIL, total bilirubin; TCM, traditional Chinese medicine; TNF-α, tumor necrosis factor α; ZDP, *Zornia diphylla* (L.) Pers.
